# Erythropoietin Gene Therapy Delays Retinal Degeneration Resulting from Oxidative Stress in the Retinal Pigment Epithelium

**DOI:** 10.3390/antiox10060842

**Published:** 2021-05-25

**Authors:** Manas R. Biswal, Zhaoyao Wang, Ryan J. Paulson, Rukshana R. Uddin, Yao Tong, Ping Zhu, Hong Li, Alfred S. Lewin

**Affiliations:** 1 Department of Pharmaceutical Sciences, Taneja College of Pharmacy, University of South Florida, Tampa, FL 33612, USA; rjpaulso@usf.edu; 2 Department of Ophthalmology, Morsani College of Medicine, University of South Florida, Tampa, FL 33612, USA; 3 Department of Internal Medicine, Morsani College of Medicine, University of South Florida, Tampa, FL 33612, USA; 4 Department of Molecular Genetics & Microbiology, College of Medicine, University of Florida, Gainesville, FL 32610, USA; zhaokekewzy@hotmail.com (Z.W.); rukshana463@ufl.edu (R.R.U.); ytong2@tulane.edu (Y.T.); l712129@ufl.edu (H.L.); lewin@UFL.EDU (A.S.L.); 5 Department of Ophthalmology, Shanghai Ninth People’s Hospital, Shanghai Jiaotong University School of Medicine, Shanghai 200011, China; 6 Department of Chemistry, University of Florida, Gainesville, FL 32603, USA; 7 Department of Cell & Molecular Biology, Tulane University, New Orleans, LA 70118, USA; 8 Department of Ophthalmology, University of Florida College of Medicine, Gainesville, FL 32610, USA; pingz@ufl.edu

**Keywords:** age related macular degeneration, oxidative stress, MnSOD, RPE, retinal degeneration, erythropoietin, gene therapy, animal model, AAV, ERG

## Abstract

Erythropoietin (EPO) plays an important role in erythropoiesis by its action in blocking apoptosis of progenitor cells and protects both photoreceptors and retinal ganglion cells from induced or inherited degeneration. A modified form of EPO, EPO-R76E has attenuated erythropoietic activity but is effective in inhibiting apoptosis, oxidative stress, and inflammation in several models of retinal degeneration. In this study, we used recombinant Adeno Associated Virus (AAV) to provide long-term sustained delivery of EPO-R76E and demonstrated its effects in a mouse model of dry-AMD in which retinal degeneration is induced by oxidative stress in the retinal pigment epithelial (RPE) cells. Experimental vector AAV-EPO-R76E and control vector AAV-GFP were packaged into serotype-1 (AAV1) to enable RPE selective expression. RPE oxidative stress-mediated retinal degeneration was induced by exon specific deletion of the protective enzyme MnSOD (encoded by *Sod2*) by cre/lox mechanism. Experimental mice received subretinal injection of AAV-EPO-R76E in the right eye and AAV-GFP in the left eye. Western blotting of RPE/choroid protein samples from AAV-EPO-R76E injected eyes showed RPE specific EPO expression. Retinal function was monitored by electroretinography (ERG). EPO-R76E over-expression in RPE delayed the retinal degeneration as measured by light microscopy in RPE specific *Sod2* knockout mice. Delivery of EPO-R76E vector can be used as a tool to prevent retinal degeneration induced by RPE oxidative stress, which is implicated as a potential cause of Age-Related Macular Degeneration.

## 1. Introduction

Age related Macular Degeneration (AMD) is one of the leading causes of permanent vision loss in people over the age of 60 [1,2]. The Retinal Pigment Epithelium (RPE) provides nutritional and metabolic support that is essential for the function of photoreceptor cells and serves as a component of the blood-retinal barrier. In the dry form of AMD, macular RPE atrophy leads to photoreceptors loss, thus affecting vision. Dysfunction and loss of RPE in AMD are associated with several genetic and environmental factors. These factors can induce oxidative stress and inflammation that play pathological roles in RPE degeneration [3]. Oxidative stress, accumulation of oxidized lipids and cholesterol, ion channel impairment, and mitochondrial dysfunction have been shown to alter RPE physiology [4,5,6,7]. Many endogenous and exogenous factors can damage mitochondrial DNA (mtDNA) in the neural retina and RPE cells resulting in reactive oxygen species (ROS) overproduction [8]. High mitochondrial ROS production imbalances antioxidant and cytoprotective systems in the RPE and play a pivotal role in AMD pathogenesis [8]. Antioxidants, growth factors, and neurotrophic factors are widely proposed to protect RPE cells from oxidative damage-associated changes [9].

Erythropoietin (EPO), a secreted cytokine, is FDA-approved for the treatment of anemia. EPO has been shown to act as a novel agent in vascular protection against acute lung injury by promoting angiogenesis [10]. EPO provides neuroprotective effects in several animal models, as it blocks apoptotic pathways and indirectly induces endogenous antioxidants in neurons [11,12]. Intravenous EPO delivery improved visual acuity and color vision in patients following indirect traumatic neuropathy [13]. Systemic or retinal delivery of EPO or EPO-R76E, a modified form of EPO with reduced erythropoietic activity, can improve the function of retinal ganglion cells and photoreceptors cells [14,15,16,17,18,19,20,21,22,23,24]. 

We reported *Sod2^flox/flox^-VMD2^cre^* mice as an animal model of dry AMD by conditional genetic deletion of manganese superoxide dismutase (MnSOD, encoded by *Sod2*), a mitochondrial antioxidant enzyme in the RPE [25]. Loss of MnSOD in the RPE leads to the induction of oxidative stress, further promoting progressive retinal degeneration seen as early as 4–6 months [26]. We have used this animal model to test various drugs, antioxidant genes, and nutritional supplements to improve the function of RPE and neural retina [27,28,29,30,31]. 

Erythropoietin and the erythropoietin receptor (EPOR) are widely expressed within retinal cells, and several groups have tested the ability of exogenous EPO to ameliorate retinal degeneration associated in animal models of diabetic retinopathy, retinitis pigmentosa, and other forms of retinal degeneration [22,32]. However, the potential of EPO to limit retinal degeneration associated with age-related macular degeneration (AMD) has not been explored. Chronic oxidative stress in the RPE plays an important role in RPE loss in dry-AMD [3,8,33,34]. In response to sustained oxidative stress, RPE cells die by necroptosis [35,36]. We hypothesize that sustained expression of EPO-R76E in the RPE using an AAV vector will improve the health and survival of RPE and retinal photoreceptors. Thus, we evaluated the efficacy of the modified form of EPO in protecting RPE from oxidative stress-induced changes in our mouse model of dry-AMD. We show that the presence of EPO-R76E slowed down the rate of functional decline and preserved retinal thickness caused by oxidative stress in the RPE.

## 2. Materials and Methods

### 2.1. Study Design

**Figure 1:**
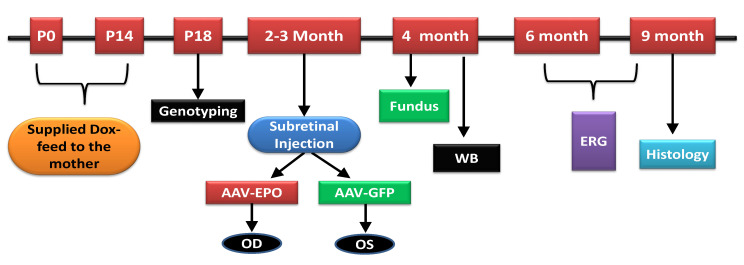
Experimental design.

EPO-R76E expression was tested in cell culture by Western blotting, and all the experiments in cell culture were performed in triplicate. A total of 10 to 12-week-old mice of both sexes (*n* = 20) were used to test the vector in vivo. The experimental vectors were delivered to the mouse eyes by subretinal injection. Three mice (*n* = 3) were analyzed for transgene expression by Western blotting 6 weeks following injection. Five mice (*n* = 4) were discarded from the study due to retinal detachment or abnormality following subretinal injection. The retinal function was recorded by scotopic ERG at 6 and 9 months of age (3 and 6 months after injection). At the end of the experiments (9 months of age), the retinal tissues were analyzed by histology. A schematic diagram of the experimental design is shown in Figure 1.

### 2.2. Plasmids and Cell Culture

The AAV plasmid having a modified form of *EPO* (EPO-R76E) cDNA was provided by Dr. Tonia S. Rex (Vanderbilt University) [23]. The Arginine residue at the 76th position of the human EPO gene (GenBank: M11319.1) coding sequence (CDS) was replaced with glutamate via site-directed mutagenesis to obtain the EPO-R76E transgene, and the transgene was cloned into an AAV plasmid. For complete gene expression, this plasmid consisted of DNA sequences of inverted tandem repeats (ITR) the human cytomegalovirus (CMV) promoter, short intron sequence, human EPO-R76E cDNA, Woodchuck Hepatitis Virus (WHV) Posttranscriptional Regulatory Element (WPRE), bovine growth hormone polyadenylation (bgh-PolyA) signal and Ampicillin resistance (Amp^r^) genes. This plasmid was used for testing EPO expression in vitro and for generating the AAV used in vivo.

Control AAV-GFP and experimental AAV-EPO-R76E plasmids were transfected to one Shot Stbl3 chemically competent *E. coli* (Thermo Scientific, Waltham, MA, USA) cells. The transfected plasmids were harvested by maxiprep and purified using cesium chloride gradient centrifugation method. HEK293T cells grown on 6 well plates were used in triplicates to transfect 4 micrograms of each plasmids using polyethylenimine (PEI) cellular transfection reagent (Polysciences, Warrington, PA, USA, catalog no: 23966-100) with a ratio of 1:2, DNA to PEI. Transfection medium with DNA and PEI was replaced with complete growth media after 24 h and further incubated for another 24 h to allow transgene expression. The next day, cells were checked for GFP fluorescence. After that, cells were dislodged using cold phosphate-buffered saline (PBS). The cells were pelleted at 14,000× *g* for 30 min at 4 °C, supernatants were collected to quantify protein concentration and kept at −80 °C for Western blotting analysis.

### 2.3. AAV Vector Production

HEK-293T Cell culture and virus processing was performed exclusively in BSL II certified biosafety cabinets at the Retinal Gene Therapy Vector Core within the University of Florida as per established protocols [37]. Briefly, one milligram of plasmids was transfected to HEK-293T cells along with helper and AAV1 serotype plasmids to produce AAV1 virus. Transfected cells were lysed by 3 freeze/thaw cycles between dry ice–ethanol and 37 °C water baths to release the AAVs. The crude lysate was clarified by iodixanol gradient centrifugation and anion exchange chromatography. Endotoxin was removed using published protocols [38]. The purified and endotoxin-free virus was diluted in Hanks’ Balanced Salt solution (HBSS) buffer, and a stock concentration of 1 × 10^13^ viral genome copies per milliliter (VG/mL) was achieved. For animal injection, the virus was diluted in HBSS buffer.

### 2.4. Animals

All the procedures involving animals in this study followed the ARVO Statement for the Use of Animals in Ophthalmic and Vision Research, and the protocols were approved by the Institutional Animal Care and Use Committee (IACUC) at the University of South Florida and the University of Florida (Approval number: IS00005958). Breeding pairs of RPE specific *Sod2* deleted mice (*Sod2^flox/flox^-VMD2-cre*) on the C57BL/6J background were set up to generate the mice for this project. These transgenic mice have the *VMD2* promoter driving inducible cre transgene [39] and loxP sites surrounding exon 3 of *Sod2* [40]. The cre transgene was induced by providing doxycycline chow (Bio-Serv) to the nursing dams of pups from P0-P14, leading to deletion of *Sod2* as described by Mao et al. [25]. The pups were screened by genotyping using the primers for *flox*, *cre*, *rd1*, *rd8*, and *rd10* (Supplementary Table S1). Since *rd1*, *rd8*, and *rd10* mutations are associated with retinal degeneration, the pups showing the mutations were removed from the study. Only the pups homozygous for the *floxed* allele of *Sod2* and transgenic for *cre* were used for this study. Intraperitoneal injection of a mixture of ketamine (95 mg/kg) and xylazine (8 mg/kg) was used to anesthetize the mice for in vivo procedures such as fundus imaging, electroretinography (ERG), and retinal injections. The procedures from one of our previous publications were followed for eye dilation and local anesthesia [28].

### 2.5. Subretinal Injections

For subretinal injection, the eyes were dilated by 1% atropine and 2.5% phenylephrine 18 h before injection. The next day, the eyes were further dilated 3 times (10 min intervals), and 1 drop of artificial tears (GenTeal, Alcon, Geneva, Switzerland) were applied to moisten the eye. Under the surgical microscope, 1 μL of 10^12^ VG/mL of AAV1-EPO-R76E (i.e., 1 × 10^9^ total viral particles) in one eye and an equal dose of AAV1-GFP in the other eye [28,41] were delivered into the subretinal space using a 33 gauge microneedle connected to 2.5 uL microsyringe. Following injection, the eyes were treated topically with antibiotics to avoid infection complications following injection and allowed mice to recover on a warm circulating water pad. The success of the subretinal injections was determined by analyzing the high-resolution imaged recorded by spectral domain coherence tomography (SD-OCT) (Supplementary Documents) 14 days after injection. Animals showing retinal detachment or any structural defect due to injection were excluded from further analysis.

### 2.6. Western Blot Analysis

Proteins from cells and retinal tissues were analyzed by Western blotting to determine the expression of modified EPO. For cell culture analysis, the cell pellet was dissolved in 100 uL of RIPA lysis buffer with protease inhibitors (Sigma, St. Louis, MO, USA, Cat no: P8340). The cells in lysis buffer were vortexed for 3 to 4 times with 10-min intervals on ice to release the protein and then centrifuged at 14,000× *g* for 30 min at 4 °C. The supernatant was collected to quantify the protein concentration. For in vivo expression studies, 1 month following subretinal delivery of vectors, the eyes were collected after euthanasia. The retina and RPE/choroid were dissected out under a surgical microscope and collected separately in 100 uL of RIPA lysis buffer with protease inhibitors. The tissues were sonicated for 30–45 s in lysis buffer while on ice, and cell debris was pelleted at 14,000× *g* for 30 min at 4 °C. Pierce™ 660 nm Protein Assay Reagent (Thermo Fisher Scientific, Cat no: 22660) was used to quantify protein concentration using the supernatant collected from cell pellets and retinal tissues. A total of 20 μg of protein were separated on SDS-PAGE gels, and proteins were transferred to PVDF membrane. The membranes were blocked with Odyssey Blocking Buffer (a phosphate-buffered saline (PBS) based formulation, Li-COR) for an hour and incubated overnight with rabbit polyclonal *Epo* primary antibody (Santa Cruz Biotechnology, Dallas, TX, USA, Cat no: sc-7956) and mouse monoclonal alpha *tubulin* (Abcam, Cambridge, UK, Cat no: ab7291) primary antibody used as a loading control. After washing in PBS-Tween 20 (0.05%) buffer, the membranes were incubated with species-specific secondary antibody (LiCor, Lincoln, NE, USA; Cat no: 92532213 and Cat no: 92668170) diluted in PBS for 1 h and washed 3 times in wash buffer (PBS-Tween 20 (0.05%)) before imaging. Labeled proteins were detected using the LiCor Clx Odyssey instrument that showed 2 different colors for 2 different protein bands depending upon size.

### 2.7. In Vivo Fundus Imaging

**Figure 2:**
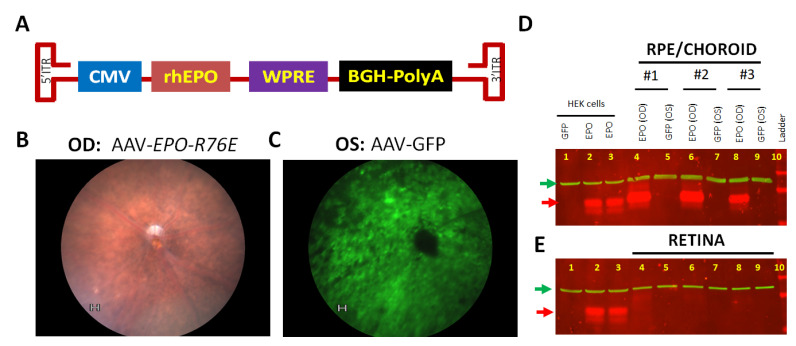
AAV-mediated protein expression in the retina. (**A**) The AAV plasmid contains rhesus EPO-R76E (rhEPO) cDNA driven by cytomegalovirus immediate early (CMV) promoter [24] and contains the Woodchuck Hepaititis Postransciptional Regulatory Element (WPRE). The vector was packaged as serotype 1 (AAV1) to promote RPE-specific transduction. The AAV-EPO-R76E experimental vector was injected in one eye of 2–3-month-old mice (**B**), and the contralateral eye was injected with control vector, AAV-GFP. One month following subretinal gene delivery GFP fluorescence (**C**) was noticed around the optic nerve by fundus imaging. Exogenous EPO-R76E was significantly increased (**D**) in the RPE/choroid of *Sod2*^flox/flox^/VMD2-cre mice (red arrow) injected with the AAV-EPO-R76E vector (lanes 4, 6 and 8) compared to eyes injected with the control AAV-GFP vector (lanes 5, 7, and 9), using an EPO specific antibody and β-actin used as a loading control (green arrow). (**E**) EPO levels were minimal (Lane 4, 6, 8) in the retinas of the same eyes. Proteins from GFP transfected HEK cells (lane 1) and EPO plasmid transfected HEK cells (Lane 2 and 3) were used in both the gels to have negative and positive control for retinal tissues.

GFP fluorescence fundus imaging was performed to check the spread and expression of the control vector (Figure 2C) using Phoenix Micron 3 fundus camera. For this, the pupils of the mice were dilated once with 1% atropine and twice with 2.5 phenylephrine, then mice were anesthetized, the cornea was lubricated by 1 drop of artificial tears (GenTeal, Alcon). The eyes of the mice were positioned to face the fundus camera, and images were recorded keeping the optic nerve at the center using GFP filter and normal bright field filter.

### 2.8. Scotopoic Electroretinography (ERG)

To monitor retinal function, the scotopic (dark-adapted) ERG response was measured using the Espion full-field ERG system Espion ColorDome Ganzfeld ERG system (Diagnosys, Inc., Lowell, MA, USA) according to an established protocol [28,29]. For this, mice were dark-adapted overnight, and pupils were dilated with 3 times (with 10 min interval) application of 1 drop each of 1% atropine and 2.5% phenylephrine. After the final step, 1 drop of a local anesthetic (proparacaine) was applied. After that, mice were anesthetized with ketamine/xyalzine as described above. A ground electrode was connected to the tail and a reference electrode was placed in the mouth. The eyes were lubricated using one drop of artificial tears (GenTeal, Alcon). Loop electrodes were placed on the eye, making sure complete connection to the surface of the eye. Once the connection was established with minimal impedance, the ERG protocol was initiated. Flashes of 20 dB (20 cds/m^2^) were applied and acquisition was performed with 1000 Hz frequency. Four sweeps were recorded with 2000 ms delay interval and average of 4 sweeps were used for ERG data analysis. The ERG a-wave, b-wave, and c-wave responses from both the eyes were recorded. The results were compared between control and experimental vector injected eyes at 3 months and 6 months following subretinal injection.

### 2.9. Light Microscopy

We used intraperitoneal injection of EUTHASOL^®^ euthanasia solution (pentobarbital sodium and phenytoin sodium) to euthanize the experimental mice. We followed the procedures for perfusions and tissue processing, embedding, and sectioning as previously described [25]. Semithin cross-sections of 0.5 μm from resin embedded retinal tissue were cut through the optic nerves and mounted on glass slides. These sections were stained with 1% toluidine blue and 2% borate in distilled water. Stained sections were examined at 4×, 20×, and 100× by light microscopy using Keyence All-in-One Fluorescence Microscope BZ-X800 (Itasca, IL, USA)

### 2.10. Statistical Analysis

GraphPad Prism 5.0 (GraphPad, San Diego, CA, USA) was used to produce the graphs. Two-tailed Student’s *t*-tests were used to test the statistical significance of differences in results. Bonferroni post hoc analysis was used to determine the true differences within the groups. All the data are represented as mean ± Standard Error of Mean (SEM) unless otherwise indicated. Significance was reported whenever the calculated *p*-value was ≤0.05. * *p* ≤ 0.05, ** *p* ≤ 0.01, *** *p* ≤ 0.001.

## 3. Results

### 3.1. EPO Expression in RPE-Specific Sod2 Deleted Mice

Even though AAV1 transduces both Müller glia and RPE following intravitreal injection, Müller glia expression is much less than the RPE [41]. In order to restrict transgene expression to RPE cells, we injected an AAV1 vector containing human-modified EPO (EPO-R76E) into the subretinal space of 3-month-old RPE-specific *Sod2* deleted mice [42]. The contralateral eyes from the same animals were injected with AAV1 expressing humanized GFP as a control to evaluate the impact of subretinal injection or virus-induced effects. By using fluorescence fundus imaging, we observed GFP expression over 50–70% of the retina (Figure 2C) that suggested the efficiency of subretinal viral delivery. To detect and quantify exogenous transgene expression, the level of AAV-delivered EPO protein expression was examined 1 month following subretinal injection using an EPO antibody. The control and experimental vector injected eyes were harvested from a cohort of mice 1 month following injection. The retina and RPE/choroid from each eye were collected separately for protein analysis. EPO antibody detected exogenous expression in RPE/choroid samples injected with AAV-EPO (Figure 2D) as we see a 37KD protein band. As expected, we found negligible expression of EPO in the retina (Figure 2E), confirming the RPE-specific tropism of AAV1 [43].

### 3.2. Improved Retinal and RPE Function

**Figure 3:**
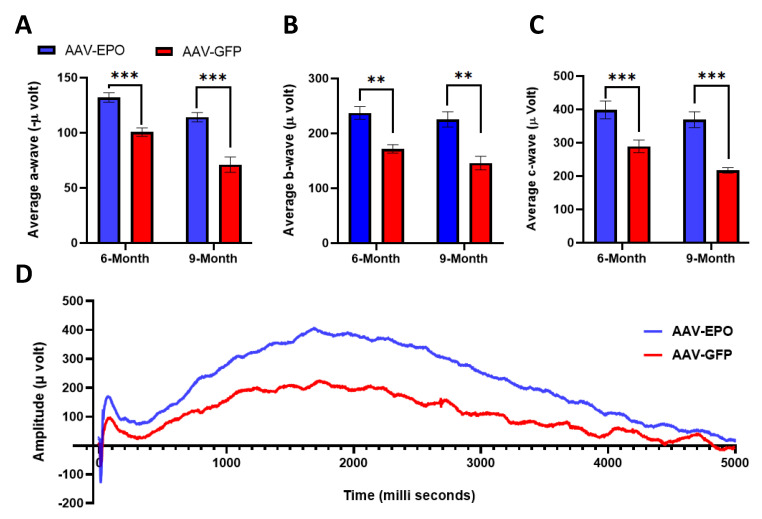
Improvement of Photoreceptor and RPE function. Dark-adapted full field electroretinogram (ERG) amplitudes measured at a light intensity of 20 cds/m^2^ at 6 months (6mo) and 9 months (9mo) of age after subretinal delivery of EPO-R76E. In the EPO treated group, significant increases in (**A**) a-wave, (**B**) b-wave and (**C**) c-wave amplitudes were recorded both at 6 months (*n* = 13) of age and 9 months of age (*n* = 11) compared to untreated group injected with GFP vector. (*p* < 0.01). Please refer to Supplementary Figure S1 for the ERG data shown as a box-and-whisker plot. (**D**) indicates the representative scotopic ERG waveforms at 9 months. ** *p* ≤ 0.01, *** *p* ≤ 0.001.

Under dark-adapted conditions, the ERG amplitudes of *Sod2* deleted mice were lower than control mice [26]. Three months following *EPO* treatment, a-wave and b-wave ERG amplitudes were significantly different between eyes treated with the experimental and the control vectors (Figure 3). At 6 months of age (3 months following injection), the eyes treated with AAV-EPO vector showed 31% improvement in a-wave response (−132.313 ± 4.337 μ volts vs. −100.654 ± 3.830 μ volts) and 38% increase in b-wave response (237.154 ± 11.829 μ volts vs. 171.692 ± 7.664 μ volts) compared to contralateral eyes injected with AAV-GFP vector (Figure 3A,B). At 9 months of age (6 months following injection), the loss in a- and b-wave response were prevented (Figure 3A,B). We found 60% more of a-wave response (114.129 ± 3.859 μ volts vs. 71.003 ± 6.398 μ volts) and 54% more of b-wave response (225.709 ± 12.856 μ volts vs. 145.791 ± 11.427 μ volts). The c-wave ERG response reflects the health of RPE. We recorded a 37% (398.692 ± 26.394 μ volts vs. 289.438 ± 18.685 μ volts) and 63% (369.282 ± 21.930 μ volts vs. 226.245 ± 12.752 μ volts) (Figure 3C) improvement in c-wave ERG responses at 6 months of age (3 months following injection) and at 9 months of age (6 months following injection), respectively, in eyes injected with AAV-EPO vector compared to untreated eyes injected with AAV-GFP vector. It should be noted that the long-term expression of GFP in the RPE of rodents does not affect the ERG response [44,45]. The representative scotopic ERG waveforms at 9 months of age is displayed in Figure 3D.

### 3.3. Improvement in Retinal Structure after Treatment with AAV-EPO-R76E Is Revealed by Light Microscopy

**Figure 4:**
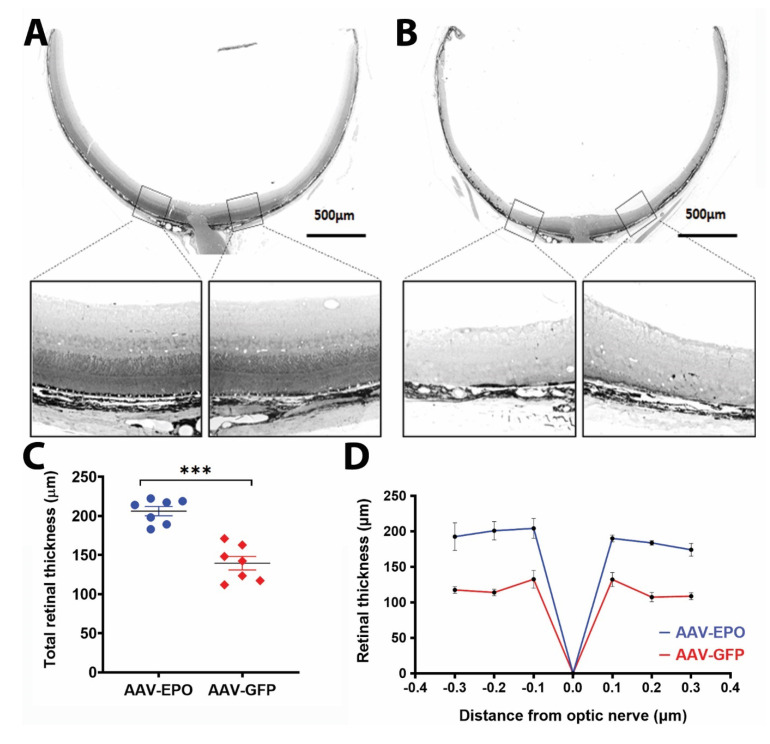
Preservation of retinal thickness: Representative low magnification and merged images of retina sections from treated (**A**) and untreated (**B**) eyes of one mouse through optic nerve and approximate areas (boxed and zoomed). (**C**) represents the measurement of the average of retinal thickness of eyes treated with AAV-EPO compared to AAV-GFP injected eyes. Spider graphs (**D**) of average retinal thickness at different distances from the optic nerve head (*n* = 7) demonstrated that AAV-EPO treatment reduced degeneration of the retina. Scale bar 500 μm. *** *p* ≤ 0.001.

**Figure 5:**
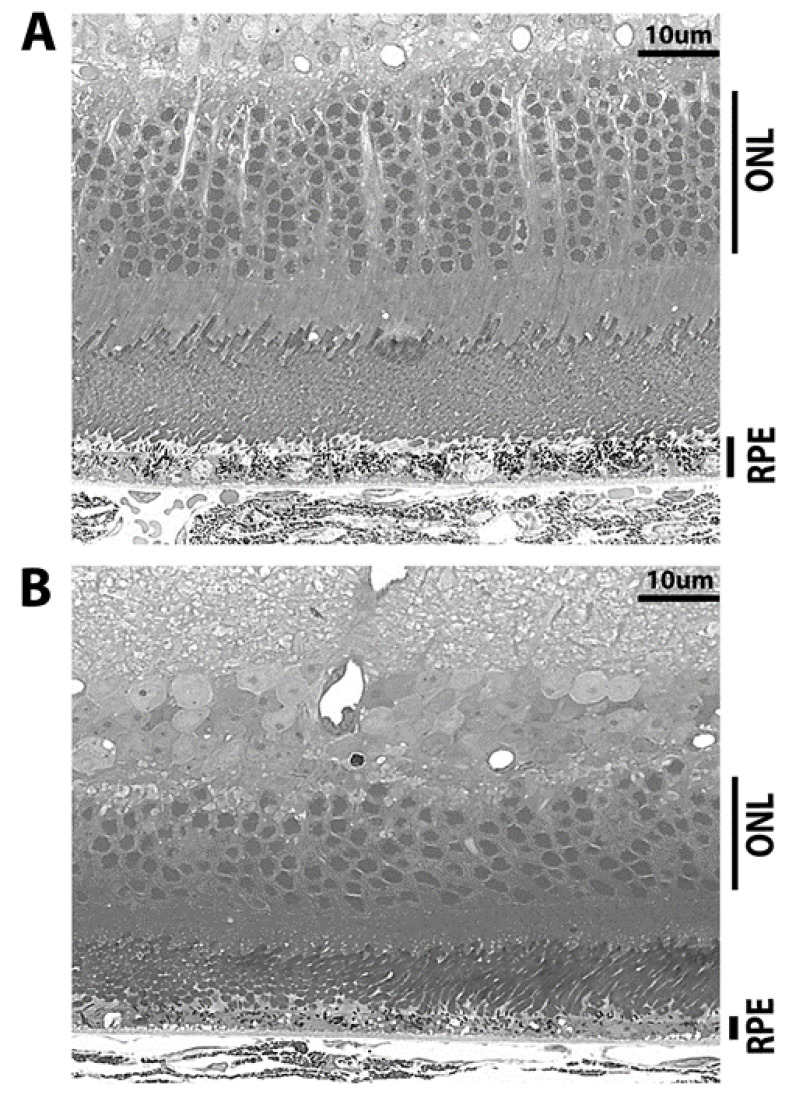
Photoreceptor and RPE preservation in AAV-EPO injected eyes. (**A**) represents the retinal sections from AAV-EPO injected eyes and (**B**) represent retinal section from AAV-GFP injected eyes. Scale bar 10 μm.

Previously, we have reported a decrease in retinal thickness in RPE-specific *Sod2* knockout mice as the age progresses [25]. The effects of *Sod2* deleted changes in the retina were visible by light microscopy as progressive RPE and photoreceptor cell degeneration in all AAV-GFP injected eyes (Figure 4A,B). The retinas of AAV-EPO treated eyes (206.195 ± 5.974 μm) were 48% thicker than the retinas of AAV-GFP treated eyes. (139.634 ± 8.630 μm) (Figure 4C). Upon quantifying the retinal thickness from the equal distance from optic nerve head ONH, we noticed the thicker retina in experimental eyes compared to control eyes (Figure 4D). In control eyes injected with AAV-GFP vector, the RPE monolayer thinning along with irregular melanin pigment distribution was noticed (Figure 5B). Thinning of the RPE monolayer is indicative of RPE loss and impaired RPE integrity. The changes in response to AAV-EPO-R76E were indicated by thicker RPE implying better structural Integrity (Figure 5A). Melanin pigment distribution was quite uniform. Rounded RPE cell nuclei were visible in AAV-EPO treated eyes, whereas RPE cell nuclei were pyknotic in untreated eyes, indicating the better health of RPE in treated eyes. The basal laminar layer in treated eyes exhibited well-preserved structure compared to GFP injected eyes. Progressive disorganization of the photoreceptor outer and inner segments and collapsed photoreceptor nuclei were indicated by the loss of outer and inner segments. More rows of photoceptor nuclei (ONL) were observed in AAV-EPO injected eyes compared to AAV-GFP injected eyes. Longer photoreceptor outer segments were seen in AAV-EPO injected eyes compared to AAV-GFP injected eyes. These results suggested that the prevention of retinal thinning predominantly occurs in the photoreceptor layer and retinal pigment epithelium (RPE).

## 4. Discussion

Clinical and experimental evidence for both dry and wet forms of age-related macular degeneration (AMD) demonstrates disruption of the structural and functional integrity of the RPE in addition to the loss of photoreceptors [3,46,47,48]. We have shown that the deletion of mitochondrial form of superoxide dismutase (MnSOD or *Sod2*), an antioxidant gene, in the RPE of mice impairs retinal structure and function. Oxidative stress is one of the key contributors to age-related retinal degeneration, particularly in dry-AMD [3,48]. Therefore, efforts are in progress to develop a therapeutic approach that can prevent further loss of structural and functional integrity of the RPE induced by oxidative damage. Growth factors offer the potential to prevent cell loss and degeneration of retinal cells from oxidative stress if they can be delivered to the specific cells. Therefore, using a cell-specific gene therapeutic approach, we have shown that subretinal delivery of serotype 1 AAV (AAV1) driving recombinant erythropoietin (EPO-R76E) can restrict EPO transgene expression in the RPE. This protects both the structural and functional integrity of RPE and retina impaired by RPE-specific oxidative stress.

Erythropoietin (EPO) is a hormone produced primarily by the kidneys, with small amounts made by the liver. EPO plays a key role in the production of red blood cells (RBCs), which carry oxygen from the lungs to the rest of the body. EPO is also expressed locally in the retina under the control of hypoxia-inducible factor (HIF-1) [49]. EPO is present in considerably higher concentrations in eyes with diabetic macular edema than in eyes with exudative AMD or normal eyes [50]. The results from several studies indicate that erythropoietic EPO is therapeutic for a broad range of neurodegenerative diseases such as axonal degeneration, peripheral nerve injury, experimental brain injury, and Alzheimer’s disease [51,52,53,54,55]. The safe use of EPO is demonstrated in the clinic with other diseases as it can traverse the intact blood–brain and blood–retina barriers in therapeutic concentrations [49,55]. It was previously reported that the systemic delivery of EPO-R76E was able to provide successful preservation of retinal ganglion cells and visual function without significantly increasing hematocrit, unlike regular EPO [16]. Tao and colleagues recently demonstrated that pre-treatment of mice by subretinal injection of AAV2-EPO, protected the retina from acute N-Methyl-N-Nitrosourea (MNU) toxicity [56]. There is a need, however, to demonstrate the best strategies for developing and delivering EPO or erythropoietic stimulating agents for the treatment of patients with atrophic or dry- AMD [57]. Adeno-associated virus (AAV) vectors can transduce a wide range of dividing and non-dividing cell types, which has made these vectors an important tool for ophthalmic gene therapy. A major advantage of AAV vectors is the long-term expression of the therapeutic gene as episomes within cells that can be obtained after in vivo gene delivery [58]. AAV-EPO gene therapy vector offers the advantages of delivering and stably expressing EPO gene (or its protein product) to the physiologically relevant target tissues such as RPE using specific AAV serotypes (AAV1) or promoters (e.g., VMD2).

Our results indicate that stable expression of EPO-R76E in RPE cells protected the RPE and its nearby photoreceptors under the conditions of oxidative stress. EPO can protect the retina by acting directly on the RPE or by acting in a paracrine fashion on photoreceptors and Müller glial. EPO has been shown to help maintain the barrier properties of the RPE, and this may contribute to its protective role [32,59]. EPO protected RPE cells barrier integrity disrupted by oxidative stress by reducing intracellular ROS and restoring cellular antioxidant potential [60]. These authors also reported that there was a reduction in the secretion of inflammatory cytokines (TNFα, and IL-1β) and a decrease in caspase-3 activity under oxidative stress in response to EPO treatment. Since EPO is secreted, it may also protect retinal structure and function by acting directly on photoreceptors. Exogenous EPO could directly interact with the photoreceptors allowing them to maintain the metabolic activity despite increased oxidative stress-related effects. This may also activate a signal transduction cascade in the photoreceptors [61,62,63,64]. The eyes from *Sod2^flox/flox^-VMD2^cre^* mice evidenced an increase in oxidative stress as early as 2 months of age. Protection from oxidative stress could be one of the reasons that EPO is permitting increased survival and prolonged function of photoreceptors. Another mechanism could be interactions with surrounding cells such as Müller glia, which, in turn, can release proteins that support the survival of photoreceptors [65,66,67].

RPE generates a series of slow potentials that can be recorded as the c-wave [68]. As these potentials can be related to specific cellular events, they provide information about RPE function and how that may be altered by disease or experimental manipulation [68]. In our study, we found significant preservation of c-wave ERG, and that could be correlated with strong RPE junctional integrity and healthy RPE [69,70]. Previously, using this model, we have seen that supplementation of daily zeaxanthin improved c-wave response compared to untreated mice [29].

In AMD patients, the RPE and photoreceptors are compromised in the macular region, causing loss of central vision. For the treatment of macular degeneration, therefore, protection of cone photoreceptors is essential because they are enriched in the macula and are critical for visual acuity. Treatment with RPE-specific EPO-R76E using gene therapy may allow patients to have a useful vision for a longer period due to extended-expression of EPO in the RPE, thus further preventing loss of vision. We observed protection of scotopic a, b, and c wave full-field ERG response signifying the protection of photoreceptors and RPE. Histological analysis at 9 months of age clearly demonstrated preservation of the photoreceptor and RPE layers (Figure 4A and Figure 5A) compared to control-treated eyes (Figure 4B and Figure 5B). In the future, we aim to perform molecular analysis of RPE/choroid and photoreceptors to evaluate changes in protective and inflammatory gene and protein expression, as shown in other studies [71]. As *Sod2* deletion is related to mitochondrial dysfunction in RPE, it will be interesting to see whether supplementation of EPO-R76E can rescue mitochondrial dysfunction and improve bioenergetics, as noticed in RPE cells derived from AMD patients [72].

EPO signaling increases choroidal macrophages and cytokine expression and exacerbates choroidal neovascularization, conditions associated with the advanced wet-form of AMD [73]. EPO receptor signaling supports retinal function after vascular injury [74], but its pro-angiogenic properties may limit the usefulness of unregulated EPO expression as a therapy for dry AMD. We plan to determine if EPO-R76E stimulated choroidal neovascularization (CNV) using the laser-induced CNV model.

This pilot study showed the protective effect of EPO in preserving retinal structure and function while maintaining stable expression in the RPE. Even though we did not see any harmful effect of EPO-R76E in our animal model of dry-AMD, we plan to study the impact of prolonged EPO expression in normal (wild type) mice. In our study, we did not measure the level of EPO expression in retinal tissue by AAV-EPO. We aim to compare the expression levels using an intravitreal or systemic injection of other EPO activating compounds or clinically approved EPO protein in further studies. One of the limitations of this study is that *Sod2* mutation is not directly implicated in the etiology of dry-AMD. However, oxidative stress is a recognized risk factor, and damage to the RPE is believed to initiate the disease process. Thus, the animal model with RPE-specific *Sod2* deletion has great potential to study therapeutic interventions.

Given that cone photoreceptor loss is responsible for central vision loss in AMD, it will be necessary to perform photopic ERG, focal ERG, and/or optokinetic responses to measure cone function and also include spectral domain optical coherence tomography (SD-OCT) to monitor progressive preservation of retinal layers in vivo [28]. Other than that, we also aim to check the contribution of different cell types (Müller glia, photoreceptors, astrocytes, microglia, endothelial cells, ganglion cells, etc.) in protecting the retina by analyzing transcriptional landscape by single-cell RNA-Seq experiments. We predict that local stable EPO expression can impact the proteome changes in RPE under conditions of oxidative stress. To enforce results obtained in this work, proteomics studies could supplement learning the proteome changes by EPO while protecting the cellular microenvironment.

## 5. Conclusions

Our study highlights for the first time the impact of EPO gene therapy in an animal model of dry AMD resulting due to oxidative stress in RPE. Additionally, this study offers the potential therapeutic options to treat RPE dysfunction resulting from chronic oxidative stress with one-time delivery. There is an opportunity to regulate the EPO expression in specific cells to eliminate the cytotoxic effects that oxidative stress causes to the retinal cells. A better understanding of the oxidative stress-related effects in the RPE and how EPO can modulate to improve the clinical outcomes needs further investigation. In the future, this will provide a great avenue to develop EPO therapy to treat dry-AMD.

## Data Availability

All the data are available through this manuscript.

## Supplementary Materials

The following are available online at https://www.mdpi.com/article/10.3390/antiox10060842/s1, Figure S1: ERG data presented using a box-and-whisker plot, whiskers indicate the 5 and 95% quantiles, Table S1: Primers used for genotyping *Sod2^flox/flox^-VMD2-cre* mice.
