# Supplementary material for: Erythropoietin Gene Therapy Delays Retinal Degeneration Resulting from Oxidative Stress in the Retinal Pigment Epithelium
Source: Antioxidants (Basel). 2021 May 25;10(6):842. doi: 10.3390/antiox10060842 (PMC8229633; doi:10.3390/antiox10060842)
Supplement: Supplementary file 1 [file antioxidants-10-00842-s001.zip › antioxidants-1197060-supplementary.pdf]

Supplementary Document.

SD-OCT imaging:

To check retinal health, two weeks following retinal injection, we used Envisu SD-OCT ophthalmic imaging system (Leica Microsystems, Buffalo Grove, IL, USA) to capture high-resolution OCT images [1,2]. Briefly, the eyes of the mice were dilated 1% atropine and 2.5% phenylephrine two to three times within 10 minutes interval. Later, the mice were anesthetized using Ketamine and Xylazine mixture as approved in the protocol. Once the mice got nonresponsive, the eyes were positioned at the frame to record the high-resolution images. The eyes with retinal detachment were discarded from the study.

References:

1. Mao, H.; Seo, S.J.; Biswal, M.R.; Li, H.; Conners, M.; Nandyala, A.; Jones, K.; Le, Y.-Z.; Lewin, A.S. Mitochondrial Oxidative Stress in the Retinal Pigment Epithelium Leads to Localized Retinal Degeneration. *Invest. Ophthalmol. Vis. Sci.* **2014**, *55*, 4613–4627, doi:10.1167/iovs.14-14633.
2. Biswal, M.R.; Han, P.; Zhu, P.; Wang, Z.; Li, H.; Ildefonso, C.J.; Lewin, A.S. Timing of Antioxidant Gene Therapy: Implications for Treating Dry AMD. *Invest. Ophthalmol. Vis. Sci.* **2017**, *58*, 1237–1245, doi:10.1167/iovs.16-21272.

Supplementary table 1.

Primers used for genotyping *Sod2* $^{flox/flox}$-VMD2-*Cre* mice

Gene		Sequence
**Flox**	**Forward Primer**	**CTTGTGACATCTGGCTGACG**
	**Reverse Primer**	**CCCAGATCTGCAATTTCCAA**
**Cre**	**Forward Primer**	**TGACGGTGGGAGAATGTTAAT**
	**Reverse Primer**	**GCCGTAAATCAATCGATGAGT**
**Rd1**	**Forward Primer-1**	**TACCCACCCTTCCTAATTTTTCTCAGC**
	**Forward Primer-2**	**GTAAACAGCAAGAGGCTTTATTGGGAAC**
	**Reverse Primer 1**	**TGACAATTACTCCTTTTCCCTCAGTCTG**
**Rd8**	**Forward Primer-1**	**GTGAAGACAGCTACAGTTCTGATC**
	**Forward Primer-2**	**GCCCCTGTTTGCATGGAGGAAACTTGGAAGACAGCTACAGTTCTTCTG**
	**Reverse Primer 1**	**GCCCCATTTGCACACTGATGAC**
**Rd10**	**Forward Primer**	**ACAAGGAACAAGGGCTCTGA**
	**Reverse Primer**	**CCTTCCACTCATTGCTAGGAC**

Supplementary figure 1:

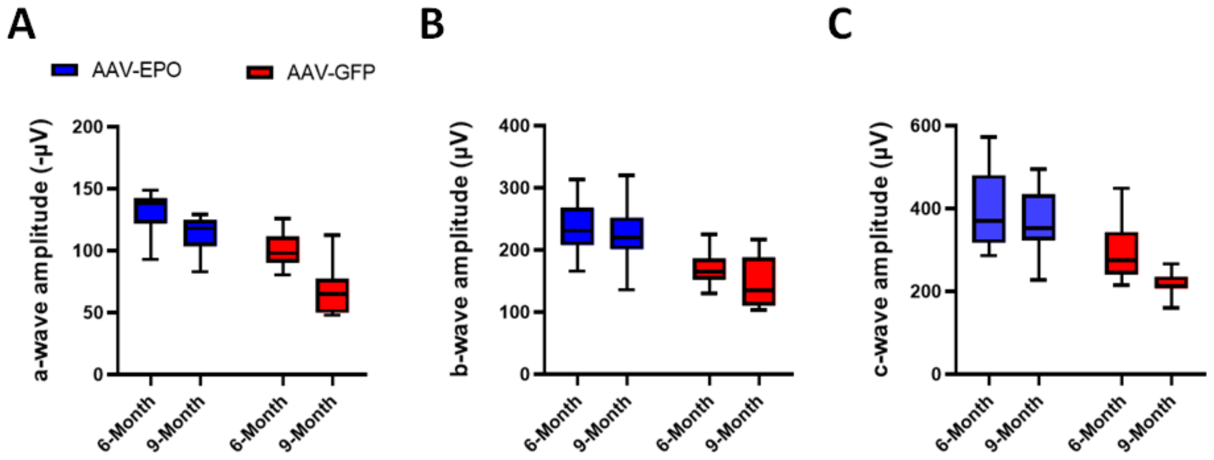

Supplementary figure-1: ERG data presented using a box-and-whisker plot, whiskers indicate the 5 and 95% quantiles.
